# A novel FIO-based hybrid piezoelectric–electromagnetic energy harvester coupled with tandem cylinders

**DOI:** 10.1038/s41598-025-32395-y

**Published:** 2025-12-18

**Authors:** Mohammadreza Rashki, Alireza Mojtahedi, Mohammad Ali Lotfollahi-Yaghin

**Affiliations:** Faculty of Civil Engineering, University of Tabriz, Tabriz, Iran

**Keywords:** Flow-induced oscillations (FIO), Vortex-induced vibration (VIV), Hydrokinetic energy harvesting, Hybrid piezoelectric-electromagnetic energy harvesters, Tandem cylinders, Energy science and technology, Engineering, Physics

## Abstract

Flow-induced oscillations (FIO) are energy-rich hydrodynamic phenomena that can be exploited to harvest renewable energy from ocean and river currents. The hydrodynamics of tandem cylinders have recently gained attention in the literature, and this study investigates a hybrid energy harvesting system based on tandem cylinder configurations. The upstream–downstream wake interference is modeled through coupled van der Pol and wake oscillators, with particular emphasis on accurately capturing both vortex-induced vibration and galloping mechanisms. Three configurations, including piezoelectric (PZT-H), electromagnetic (EMT-H), and a new proposed hybrid piezoelectric–electromagnetic coupled with tandem cylinders (HEPT-H), are analyzed under varying spacing ratios and reduced velocities. Findings highlight that galloping is the dominant instability driving large-amplitude responses, and its proper modeling is critical for predicting and maximizing harvested energy. The proposed HEPT-H system takes advantage of this mechanism, nearly doubling the harvested power and improving efficiency by about 20% compared with single-harvester systems. A multi-criteria decision-making method (TOPSIS) was employed to rank the harvesters under different cylinder spacing configurations according to their relative closeness to the ideal solution. The HEPT-H system with a center-to-center cylinder spacing of four diameters indicated the best performance, achieving a maximum output of 0.071 W and a peak efficiency of 69.78%. This research emphasizes the significant potential of HEPT-H systems in FIO and demonstrates that tandem configurations outperform isolated cylinders, underscoring their effectiveness for advancing sustainable hydrokinetic energy applications.

## Introduction

Renewable and environmentally friendly energy sources are in global demand nowadays. This necessity arises because of concerns about climate change, fossil fuel depletion, and the need for energy security. Among the diverse clean energy sources, oceanic, river, and wind currents have emerged as particularly promising. The advantages of flow currents are resource availability, environmental cleanliness, long-term sustainability, and economic viability. Oceanic, river, and wind currents can operate continuously in various geographic regions. Also, these systems provide a reliable and scalable way to meet rising energy demands. With ongoing advances in technology, energy from oceans, rivers, and wind is set to become a key part of the global transition to a low-carbon future.

Flow-induced oscillation (FIO) is a natural phenomenon that can be considered as an energy source from the ocean, rivers, and wind currents. FIO occurs when flow passes a bluff body, like a cylinder. Energy harvesting of FIO from a single cylinder for the first time was introduced by Bernitsas et al.^1^. Since then, several researchers have reported how different parameters could improve or worsen^2–4^ the value of energy harvesting from FIO. Comprehensive experimental findings and analyses related to FIO can be found in the reviews by Lv et al.^5^, Rashki et al.^6^, Ma and Zhou^7^, Rostami et al.^8^, and Wang et al.^9^.

Vortex-induced vibration (VIV) and galloping are two primary forms of FIO used for energy harvesting^10–13^. VIV arises from periodic vortex shedding around bluff bodies, such as a single circular cylinder, and normally results in limited-amplitude, frequency-locked oscillations. In contrast, galloping is a self-excited instability that occurs above a critical flow velocity, with oscillation amplitude increasing without bound as velocity rises. Compared to VIV, galloping can yield higher energy output but is more sensitive to flow conditions and structural instability^14–17^. While VIV and galloping are frequently discussed separately, the transition between them plays an important role in tandem-cylinder dynamics. At low and moderate reduced velocities, the downstream cylinder primarily undergoes VIV, dominated by periodic vortex shedding and lock-in between the vortex-shedding and natural frequencies. As the flow velocity increases, the wake deficit generated by the upstream cylinder alters the mean and fluctuating lift on the downstream cylinder. When the displacement-dependent lift slope becomes positive, the forces inject energy into the oscillation, resulting in a self-excited instability characteristic of galloping. This transition from frequency-locked VIV to broadband galloping leads to a substantial increase in vibration amplitude, and therefore, in the mechanical energy available for harvesting. The present study explicitly examines this shift and quantifies the dominant mechanism across flow-speed ranges and spacing ratios.

Abdelkefi et al.^18,19^ explored energy harvesting from galloping using piezoelectric harvesters. Sun et al.^20^ investigated piezoelectric energy harvesting from FIO, specifically considering both VIV and galloping in non-circular cylinders. Ding et al.^21^ conducted a similar study utilizing both VIV and galloping to harvest energy from square, triangular, and quasi-trapezoidal cylinders. Also, Andrianne et al.^22^ examined galloping-based energy harvesting from a square cylinder through wind tunnel experiments. Moreover, passive turbulent control has also been employed as an effective strategy to enhance galloping-based energy harvesting^23–25^. However, the long-term performance and reliability of such systems require careful consideration, as they are susceptible to biofouling and marine growth, which can significantly degrade their efficiency and functionality over time^26–28^.

While commonly associated with non-circular cross-sections, galloping can also occur in tandem cylinder arrangements due to wake-body interactions. Compared to a single circular cylinder, the FIO of multiple cylinders in cross-flow exhibits considerably greater complexity. This is because of wake interference, coupling effects, and altered vortex shedding dynamics^29–31^. Bokaian and Geoola^32^ evaluated the dynamic response of a downstream cylinder placed behind a fixed upstream cylinder, identifying various excitation mechanisms, including vortex-induced resonance, galloping, combined vortex resonance and galloping, as well as distinct occurrences of each phenomenon. Furthermore, their study highlighted the influence of key parameters such as mass ratio (m^*^), damping ratios (*ζ*), and spacing ratio (defined as the center-to-center distance between the cylinders normalized by the cylinder diameter) on the oscillatory behavior of the downstream cylinder.

Hu et al.^33^ investigated the effects of varying m^*^ and ζ on downstream cylinders arranged in tandem within a wind tunnel. Their findings indicated that an increase in the combined m^*^ζ leads to a progressive reduction in both vibration amplitude and the extent of the vibration region. Similarly, Assi et al.^34^ conducted experiments in a water tunnel and observed galloping behavior in the downstream cylinder, reporting a consistent trend of decreasing vibration amplitude with increasing m^*^ζ. Also, Assi et al.^35^ demonstrated that increasing the spacing ratio between cylinders results in a reduction of vibration amplitude, in some cases yielding amplitudes even lower than those observed for a single isolated cylinder. Tamimi et al.^36^ investigated the effects of upstream sharp-edged square and diamond-shaped cylinders on the FIO of a downstream circular cylinder. Their results revealed that the wake generated by the upstream sharp-edged cylinder significantly reduced the lift force coefficient on the downstream cylinder by approximately 40%.

The use of FIO, including galloping and VIV, has attracted significant attention in recent years as a potential source of renewable energy. Zhang et al.^37^ investigated piezoelectric energy harvesting from a downstream cylinder in a tandem configuration within a wind tunnel. Similarly, Chen et al.^38^ explored electromagnetic energy harvesting from a downstream tandem cylinder under wind tunnel conditions. In water tunnel experiments, which are the focus of the present study, several investigations have also explored energy harvesting from FIO. Tamimi et al.^39^ evaluated the influence of upstream square, diamond, and circular cylinders on the performance of a downstream circular cylinder. Their results demonstrated that wake interference from dissimilar upstream shapes can significantly enhance the mechanical power output of the circular oscillator by approximately 200%. Tamimi et al.^40^ calculated electromagnetic energy harvesting from free-free and fixed-free square cylinders. The results indicated that double tandem square cylinders improve the maximum efficiency of the energy harvester by 97% as compared to two separate isolated oscillators.

Sun et al.^41^ evaluated energy harvesting from two free-free cylinders with passive turbulent control (PTC). PTC could add advantages to harvest energy from galloping for both circular cylinders. Ding et al.^42,43^ conducted a numerical investigation of free-free tandem cylinders with PTC and observed a characteristic 2 S (two single vortices) shedding pattern in the initial branch of VIV, while both 2P (two pairs of vortices) and 2P + 2 S modes were identified in the upper branch. Furthermore, researchers have investigated the use of three cylinders equipped with PTC in a tandem configuration to enhance energy harvesting in FIO-based systems^44–46^. However, Zeinoddini et al.^47^ demonstrated that the performance of tandem cylinders can deteriorate when subjected to marine fouling in real marine environments.

Various energy harvesters have been developed for FIO-based systems, with piezoelectric^48–51^, electromagnetic^52–54^, and triboelectric^55–57^ transducers being the most commonly employed mechanisms. While numerous studies have explored these individual transduction mechanisms, the application of hybrid energy harvesting systems, particularly for galloping-based energy, has received comparatively limited attention. Lai et al.^58^ investigated a hybrid piezo-dielectric wind energy harvester designed for VIV-based energy conversion. Similarly, Muthalif et al.^59^ proposed a hybrid piezoelectric–electromagnetic water energy harvester utilizing a dual-mass system for VIV applications. In another study, Muthalif et al.^60^ evaluated a hybrid piezoelectric–electromagnetic harvester based on the VIV of a rotational cylinder for water flow energy extraction. In addition, Hasheminejad et al.^61^ investigated a dual-functional hybrid electromagnetic–piezoelectric energy harvester designed for pipeline-based VIV systems, demonstrating the potential of hybrid approaches in enhancing energy conversion efficiency.

Therefore, this study focuses on the hybrid electromagnetic–piezoelectric energy harvester coupled with free–free tandem cylinders (HEPT-H), with particular attention to galloping regimes where energy conversion is most significant. While previous research has primarily examined single-harvester mechanisms on tandem cylinders, the potential of HEPT-H configurations remains largely unexplored. To address this gap, this study develops and evaluates a nonlinear wake-deficit oscillator model to quantify how HEPT-H systems can enhance energy efficiency compared to individual piezoelectric (PZT-H) and electromagnetic harvesters (EMT-H). Furthermore, by employing a statistical ranking method (TOPSIS), this study provides a comprehensive comparison of harvester performance across different spacing ratios, thereby clarifying the relative advantages between hybrid and single harvester approaches.

## Methodology

This study adopts and modifies the wake-deficit oscillator framework originally proposed by Soares and Srinil^62^ to propose a mathematical model to investigate the energy harvesting of two identical circular cylinders arranged in a tandem configuration, both undergoing transverse FIO. The modeling framework focuses on the wake interference regime and introduces a nonlinear oscillator system that accounts for FIO mechanisms acting on the downstream cylinder. The governing equations are developed based on coupled wake-deficit and van der Pol oscillators, considering time-varying hydrodynamic interactions between the cylinders.

### Modeling assumptions and configuration

The following assumptions and configurations were applied:

- Both cylinders are assumed to possess identical structural characteristics, including diameter, mass, stiffness coefficients, and natural frequencies in both air and quiescent water. The analysis focuses on the influence of varying spacing ratios, reduced flow velocities $\:\left({U}^{*}\right)$, Reynolds numbers (Re), and mass ratios $\:\left({m}^{*}\right)$ on the system’s dynamic response.
- The spacing ratio between the two cylinders exceeds three, representing the wake interference regime^63^, where the motion of the upstream cylinder affects the downstream cylinder, but not the reverse^64–66^.
- The wake interference threshold was defined as spacing ratios > 3, following the classical wake-interference regime identified in experiments and numerical analyses. In this spacing range, the wake of the upstream cylinder strongly influences the downstream flow, whereas the upstream cylinder remains largely unaffected. This ensures that the modeled wake–deficit interactions are physically realistic and consistent with observed tandem-cylinder flow behavior.
- The upstream cylinder is subject to a steady uniform flow, while the downstream cylinder is exposed to a spatially and temporally varying velocity field due to wake shielding effects.
- The free–free boundary condition was adopted to idealize the cylinders as freely oscillating structures without end restraints. This condition minimizes stiffness and constraint effects, allowing the flow-induced oscillations to be governed primarily by fluid–structure interactions rather than boundary rigidity.
- The flow Reynolds number varies approximately within 1.5 × 10^3^≲Re ≲ 2.1 × 10^4^. According to Sumer^67^, this range corresponds to the subcritical regime, in which the boundary layer along the cylinder surface remains laminar while the wake exhibits transitional or weakly turbulent characteristics with periodic vortex shedding. Therefore, the modeled flow represents a laminar–transitional wake regime, rather than a fully developed turbulent state.

### Wake-deficit characterization

To describe the wake generated by the upstream cylinder, a wake-deficit model is employed based on the similarity solution of the boundary layer theory. The wake velocity profile is formulated as^62^:

1 $$\:X\left(d,{\delta\:}_{y}\right)=1-{\left(\frac{{C}_{D1}}{d}\right)}^{1/2}\left[\alpha\:{e}^{-\frac{{\gamma\:}^{2}}{4}}+\beta\:{e}^{-\frac{{\gamma\:}^{2}}{4}}{\int\:}_{0}^{\gamma\:}{e}^{\frac{{\varnothing\:}^{2}}{4}}d\varnothing\:\right]$$

$$\:d=\frac{S}{D}$$

$$\:{\delta\:}_{y}=\frac{\mathrm{d}\mathrm{i}\mathrm{s}\mathrm{p}\mathrm{l}\mathrm{a}\mathrm{c}\mathrm{e}\mathrm{m}\mathrm{e}\mathrm{n}\mathrm{t}\:\mathrm{o}\mathrm{f}\:\mathrm{d}\mathrm{o}\mathrm{w}\mathrm{n}\mathrm{s}\mathrm{t}\mathrm{r}\mathrm{e}\mathrm{a}\mathrm{m}\:\mathrm{c}\mathrm{y}\mathrm{l}\mathrm{i}\mathrm{n}\mathrm{d}\mathrm{e}\mathrm{r}-\mathrm{d}\mathrm{i}\mathrm{s}\mathrm{p}\mathrm{l}\mathrm{a}\mathrm{c}\mathrm{e}\mathrm{m}\mathrm{e}\mathrm{n}\mathrm{t}\:\mathrm{o}\mathrm{f}\:\mathrm{u}\mathrm{p}\mathrm{s}\mathrm{t}\mathrm{r}\mathrm{e}\mathrm{a}\mathrm{m}\:\mathrm{c}\mathrm{y}\mathrm{l}\mathrm{i}\mathrm{n}\mathrm{d}\mathrm{e}\mathrm{r}}{D}$$

where $\:X$ is the normalized wake velocity. $\:{C}_{D1}$ is the mean drag coefficient of the upstream cylinder. $\:d$ is the non-dimensional spacing ratio. $\:{\delta\:}_{y}$ is the dimensionless staggered position between cylinders. $\:\alpha\:$ and $\:\beta\:$ are empirical wake profile coefficients. The similarity variable $\:\gamma\:$ governs the shape of the wake profile and is expressed as:

2 $$\:\gamma\:\left(d,{\delta\:}_{y}\right)={\delta\:}_{y}{\left(\frac{4\lambda\:}{{C}_{D1}}\right)}^{1/2}$$

where $\:\lambda\:$ is the empirical wake profile coefficient. The wake-induced in-line and transverse force coefficients acting on the downstream cylinder are derived based on this velocity deficit:

3 $$\:{C}_{D12}\left(d,{\delta\:}_{y}\right)={X}^{2}\left(d,{\delta\:}_{y}\right){C}_{D2}$$

4 $$\:{C}_{L12}\left(d,{\delta\:}_{y}\right)=-\frac{4}{\sqrt{\lambda\:d}}X\left(d,{\delta\:}_{y}\right)\left[\frac{\gamma\:}{2}\left(\alpha\:{e}^{-\frac{{\gamma\:}^{2}}{4}}+\beta\:{e}^{-\frac{{\gamma\:}^{2}}{4}}{\int\:}_{0}^{\gamma\:}{e}^{\frac{{\varnothing\:}^{2}}{4}}d\varnothing\:\right)-\beta\:{e}^{-\frac{{\gamma\:}^{2}}{4}}\right]{C}_{D2}$$

These expressions quantify the flow-induced forces acting on the downstream structure and are critical to capturing the nonlinear interaction in the coupled oscillator system.

### Governing equations of motion

**Fig. 1:**
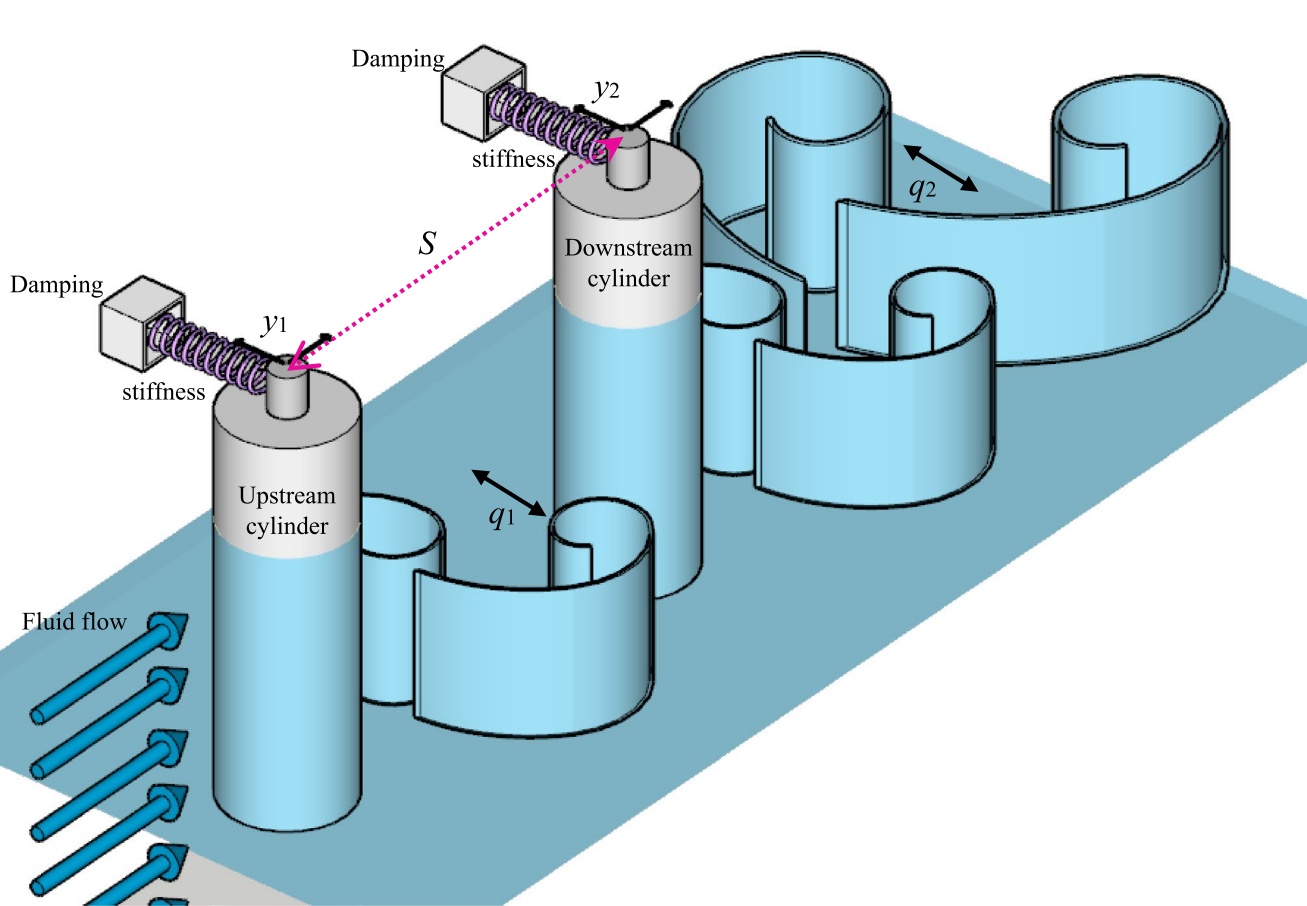
Schematic view of tandem cylinders’ arrangement (free–free condition). The upstream and downstream cylinders are aligned with flow velocity and separated by center-to-center spacing (*S*). The spacing ratio *d* governs wake interference effects that influence flow-induced oscillations (FIO) and energy harvesting response.

The cross-flow motion of each cylinder (Fig. 1) is governed by a second-order differential equation that includes both structural and hydrodynamic forces. The VIV of each cylinder is modeled using a forced van der Pol oscillator coupled with equations for the cylinder acceleration. The complete set of dimensionless equations is expressed as^62^:

Upstream cylinder (VIV):

5 $$\:{\ddot{y}}_{1}+\left(2\xi\:+\frac{{C}_{D1}}{2\mu\:}\sqrt{\frac{{{U}^{*}}^{2}}{4{\pi\:}^{2}}+{{\dot{y}}_{1}}^{2}}\right){\dot{y}}_{1}+{y}_{1}=\left(\sqrt{\frac{{{U}^{*}}^{2}}{4{\pi\:}^{2}}+{{\dot{y}}_{1}}^{2}}\right)\frac{{U}^{*}{C}_{L01}}{8\pi\:\mu\:}{q}_{1}$$

6 $$\:{\ddot{q}}_{1}+{\in\:}_{1}{St}_{1}{U}^{*}\left({{q}_{1}}^{2}-1\right){\dot{q}}_{1}+{\left({St}_{1}{U}^{*}\right)}^{2}{q}_{1}={A}_{1}{\ddot{y}}_{1}$$

Downstream cylinder (VIV + galloping):

7 $$\:{\ddot{y}}_{2}+\left(2\xi\:+\frac{{C}_{D2}}{2\mu\:}\sqrt{\frac{{{{X}^{2}U}^{*}}^{2}}{4{\pi\:}^{2}}+{{\dot{y}}_{2}}^{2}}\right){\dot{y}}_{2}+{y}_{2}=\frac{{{U}^{*}}^{2}{C}_{L12}}{8{\pi\:}^{2}\mu\:}+\left(\sqrt{\frac{{{{X}^{2}U}^{*}}^{2}}{4{\pi\:}^{2}}+{{\dot{y}}_{2}}^{2}}\right)\frac{{U}^{*}{C}_{L02}}{8\pi\:\mu\:}{q}_{2}$$

8 $$\:{\ddot{q}}_{2}+{\in\:}_{2}{St}_{2}{U}^{*}X\left({{q}_{2}}^{2}-1\right){\dot{q}}_{2}+{\left({St}_{2}{U}^{*}X\right)}^{2}{q}_{2}={A}_{2}{\ddot{y}}_{2}$$

where $\:{y}_{1}$ is dimensionless (displacement/*D*) cross-flow displacements of the upstream cylinder and $\:{y}_{2}$ is the dimensionless cross-flow displacements of the downstream cylinder. $\:{q}_{1}$ and $\:{q}_{2}$ are wake oscillator variables. $\:\mu\:$ is the reduced effective mass. $\:{St}_{1}$ and $\:{St}_{2}$ are the Strouhal numbers of the downstream and upstream cylinders, respectively. $\:{\in\:}_{1}$, $\:{\in\:}_{2}$, $\:{A}_{1}$ and $\:{A}_{2}$ are empirical coefficients for the wake oscillators. It is worth noting that the single-cylinder configuration can be modeled using Eqs. (5) and (6), which represent the fundamental governing equations for VIV in an isolated cylinder.

### Solid-fluid-piezoelectric coupling (PZT-H)

**Fig. 2:**
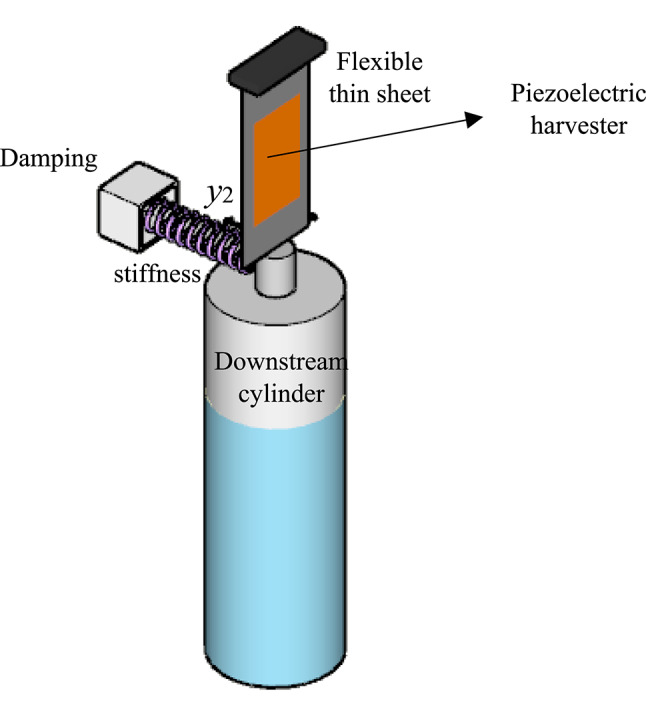
Schematic view of the PZT-H coupled with the downstream cylinder. The fluid–structure–piezoelectric coupling converts transverse oscillations into voltage and power output.

A constitutive equation, following the framework established by Mehmood et al.^68^, is employed to model the electrical coupling with the FIO oscillator. The coupled governing equations describing the interaction between the structural dynamics, fluid flow, and piezoelectric transducer (solid–fluid–piezoelectric interaction) for the downstream cylinder energy harvester (Fig. 2) are formulated as follows:

Downstream cylinder (VIV + galloping + piezoelectric coupling):

9 $$\:{\ddot{y}}_{2}+\left(2\xi\:+\frac{{C}_{D2}}{2\mu\:}\sqrt{\frac{{{{X}^{2}U}^{*}}^{2}}{4{\pi\:}^{2}}+{{\dot{y}}_{2}}^{2}}\right){\dot{y}}_{2}+{y}_{2}-{v}_{p}=\frac{{{U}^{*}}^{2}{C}_{L12}}{8{\pi\:}^{2}\mu\:}+\left(\sqrt{\frac{{{{X}^{2}U}^{*}}^{2}}{4{\pi\:}^{2}}+{{\dot{y}}_{2}}^{2}}\right)\frac{{U}^{*}{C}_{L02}}{8\pi\:\mu\:}{q}_{2}$$

10 $$\:{\ddot{q}}_{2}+{\in\:}_{2}{St}_{2}{U}^{*}X\left({{q}_{2}}^{2}-1\right){\dot{q}}_{2}+{\left({St}_{2}{U}^{*}X\right)}^{2}{q}_{2}={A}_{2}{\ddot{y}}_{2}$$

11 $$\:{\dot{v}}_{p}+{\sigma\:}_{2,p}{v}_{p}+{\sigma\:}_{1,p}{\dot{y}}_{2}=0$$

where $\:{v}_{p}$ is dimensionless electric tensions, $\:{\sigma\:}_{1,p}$ and $\:{\sigma\:}_{2,p}$ dimensionless quantities related to the PZT-H are defined as:

12 $$\:{V}_{p}=\frac{{M{\omega\:}^{2}Dv}_{p}}{{\theta\:}_{p}}$$

13 $$\:{\sigma\:}_{1,p}=\frac{{{\theta\:}_{p}}^{2}}{{C}_{p}M{\omega\:}^{2}}$$

14 $$\:{\sigma\:}_{2,p}=\frac{1}{{C}_{p}{R}_{p}\omega\:}$$

where $\:{V}_{p}$ is the electric tension of the PZT-H circuit and $\:{\theta\:}_{p}$ is the piezoelectric-mechanical coupling constant. $\:M$ is the total effective mass. $\:\omega\:$ is the natural angular frequency. $\:{C}_{p}$ is piezoelectric capacitance and $\:{R}_{p}$ is the coil load loss of the PZT-H circuit.

The governing equations for the piezoelectric energy harvester coupled with an isolated cylinder (PZI-H) can be expressed as follows:

15 $$\:{\ddot{y}}_{1}+\left(2\xi\:+\frac{{C}_{D1}}{2\mu\:}\sqrt{\frac{{{U}^{*}}^{2}}{4{\pi\:}^{2}}+{{\dot{y}}_{1}}^{2}}\right){\dot{y}}_{1}+{y}_{1}-{v}_{p}=\left(\sqrt{\frac{{{U}^{*}}^{2}}{4{\pi\:}^{2}}+{{\dot{y}}_{1}}^{2}}\right)\frac{{U}^{*}{C}_{L01}}{8\pi\:\mu\:}{q}_{1}$$

16 $$\:{\ddot{q}}_{1}+{\in\:}_{1}{St}_{1}{U}^{*}\left({{q}_{1}}^{2}-1\right){\dot{q}}_{1}+{\left({St}_{1}{U}^{*}\right)}^{2}{q}_{1}={A}_{1}{\ddot{y}}_{1}$$

17 $$\:{\dot{v}}_{p}+{\sigma\:}_{2,p}{v}_{p}+{\sigma\:}_{1,p}{\dot{y}}_{2}=0$$

The damping induced by the piezoelectric subsystem comprises two components: mechanical damping from the pulley–belt transmission and piezoelectric damping (*C*_pz_​). The former is embedded in the system identification through the damping ratio ($\:\xi\:$), while the latter (*C*_pz_​) represents a virtual damping effect due to the electromotive force generated during power conversion. This additional term is defined as $\:{C}_{pz}=-\frac{{{\theta\:}_{p}}^{2}}{{R}_{p}}$ and inherently incorporated into the governing dynamics via the coupled electromagnetic equations (Eqs. (11) and (17)).

### Solid-fluid-electromagnetic coupling (EMT-H)

**Fig. 3:**
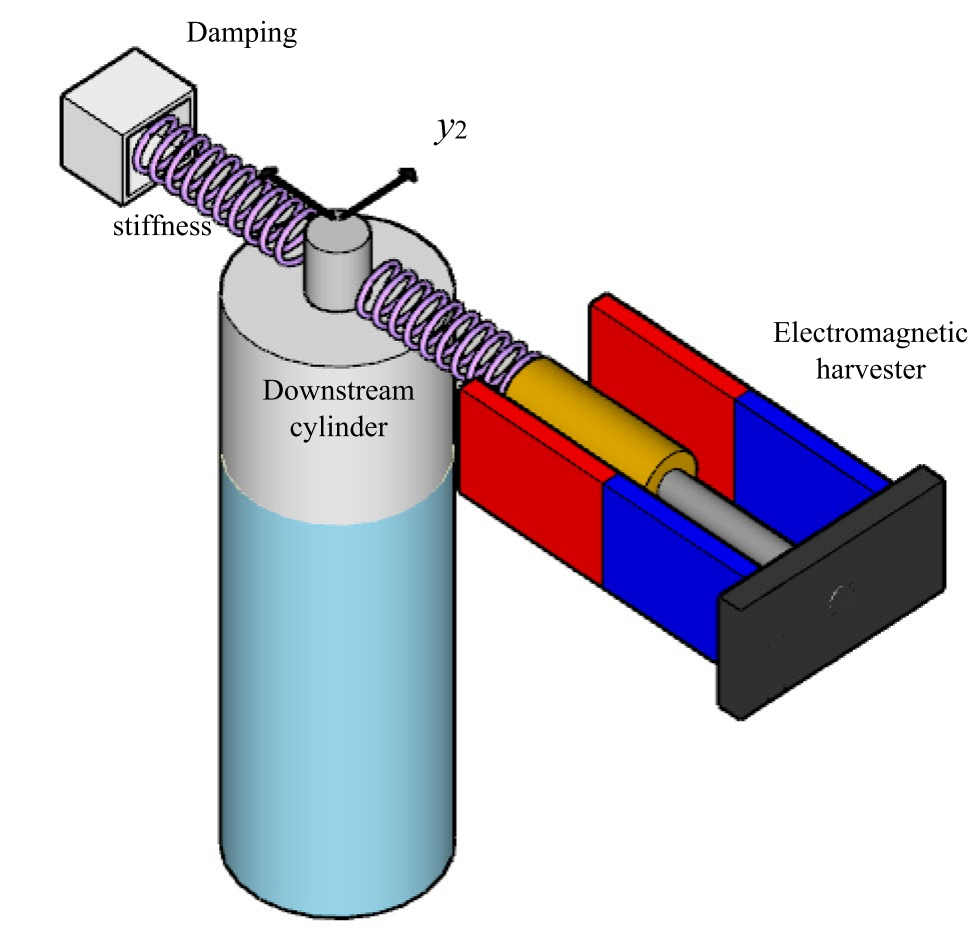
Schematic view of the EMT-H coupled with the downstream cylinder. Cylinder motion induces an electric current through electromagnetic coupling, producing harvested power.

Similar to the previous section, electromagnetic coupling could be employed to model the electrical coupling (Fig. 3) with the FIO oscillator^3^ as follows:

Downstream cylinder (VIV + galloping + electromagnetic coupling):

18 $$\:{\ddot{y}}_{2}+\left(2\xi\:+\frac{{C}_{D2}}{2\mu\:}\sqrt{\frac{{{{X}^{2}U}^{*}}^{2}}{4{\pi\:}^{2}}+{{\dot{y}}_{2}}^{2}}\right){\dot{y}}_{2}+{y}_{2}-{i}_{e}=\frac{{{U}^{*}}^{2}{C}_{L12}}{8{\pi\:}^{2}\mu\:}+\left(\sqrt{\frac{{{{X}^{2}U}^{*}}^{2}}{4{\pi\:}^{2}}+{{\dot{y}}_{2}}^{2}}\right)\frac{{U}^{*}{C}_{L02}}{8\pi\:\mu\:}{q}_{2}$$

19 $$\:{\ddot{q}}_{2}+{\in\:}_{2}{St}_{2}{U}^{*}X\left({{q}_{2}}^{2}-1\right){\dot{q}}_{2}+{\left({St}_{2}{U}^{*}X\right)}^{2}{q}_{2}={A}_{2}{\ddot{y}}_{2}$$

20 $$\:\frac{d{i}_{e}}{dt}+{\sigma\:}_{2,e}{i}_{e}+{\sigma\:}_{1,e}{\dot{y}}_{2}=0$$

where $\:i$ is the dimensionless electric current, $\:{\sigma\:}_{1,e}$ and $\:{\sigma\:}_{2,e}$ are the dimensionless quantities related to the EMT-H are defined as:

21 $$\:{I}_{e}=\frac{M{\omega\:}^{2}D{i}_{e}}{{\theta\:}_{e}}$$

22 $$\:{\sigma\:}_{1,e}=\frac{{{\theta\:}_{e}}^{2}}{{L}_{c}M{\omega\:}^{2}}$$

23 $$\:{\sigma\:}_{2,e}=\frac{{R}_{e}}{{L}_{c}\omega\:}$$

where $\:{I}_{e}$ is the electric current of the EMT-H circuit, and $\:{\theta\:}_{e}$ is the electromagnetic-mechanical coupling constant. $\:\omega\:$ is the natural angular frequency. $\:{L}_{c}$ is the electromagnetic coil inductance and $\:{R}_{e}$ is the coil load loss of the EMT-H circuit.

The equations describing the electromagnetic energy harvester coupled with an isolated cylinder (EMI-H) can be formulated as follows:

24 $$\:{\ddot{y}}_{1}+\left(2\xi\:+\frac{{C}_{D1}}{2\mu\:}\sqrt{\frac{{{U}^{*}}^{2}}{4{\pi\:}^{2}}+{{\dot{y}}_{1}}^{2}}\right){\dot{y}}_{1}+{y}_{1}-{i}_{e}=\left(\sqrt{\frac{{{U}^{*}}^{2}}{4{\pi\:}^{2}}+{{\dot{y}}_{1}}^{2}}\right)\frac{{U}^{*}{C}_{L01}}{8\pi\:\mu\:}{q}_{1}$$

25 $$\:{\ddot{q}}_{1}+{\in\:}_{1}{St}_{1}{U}^{*}\left({{q}_{1}}^{2}-1\right){\dot{q}}_{1}+{\left({St}_{1}{U}^{*}\right)}^{2}{q}_{1}={A}_{1}{\ddot{y}}_{1}$$

26 $$\:\frac{d{i}_{e}}{dt}+{\sigma\:}_{2,e}{i}_{e}+{\sigma\:}_{1,e}{\dot{y}}_{2}=0$$

The damping induced by the electromagnetic subsystem comprises two components: mechanical damping from the pulley–belt transmission and electromagnetic damping (*C*_em_​). The former is embedded in the system identification through the damping ratio ($\:\xi\:$), while the latter (*C*_em_​) represents a virtual damping effect due to the electromotive force generated during power conversion. This additional term is defined as $\:{C}_{pz}=-\frac{{{\theta\:}_{e}}^{2}}{{R}_{p}}$ and inherently incorporated into the governing dynamics via the coupled electromagnetic equations (Eqs. (20) and (26)).

### Solid-fluid-piezoelectric-electromagnetic coupling (HEPT-H)

**Fig. 4:**
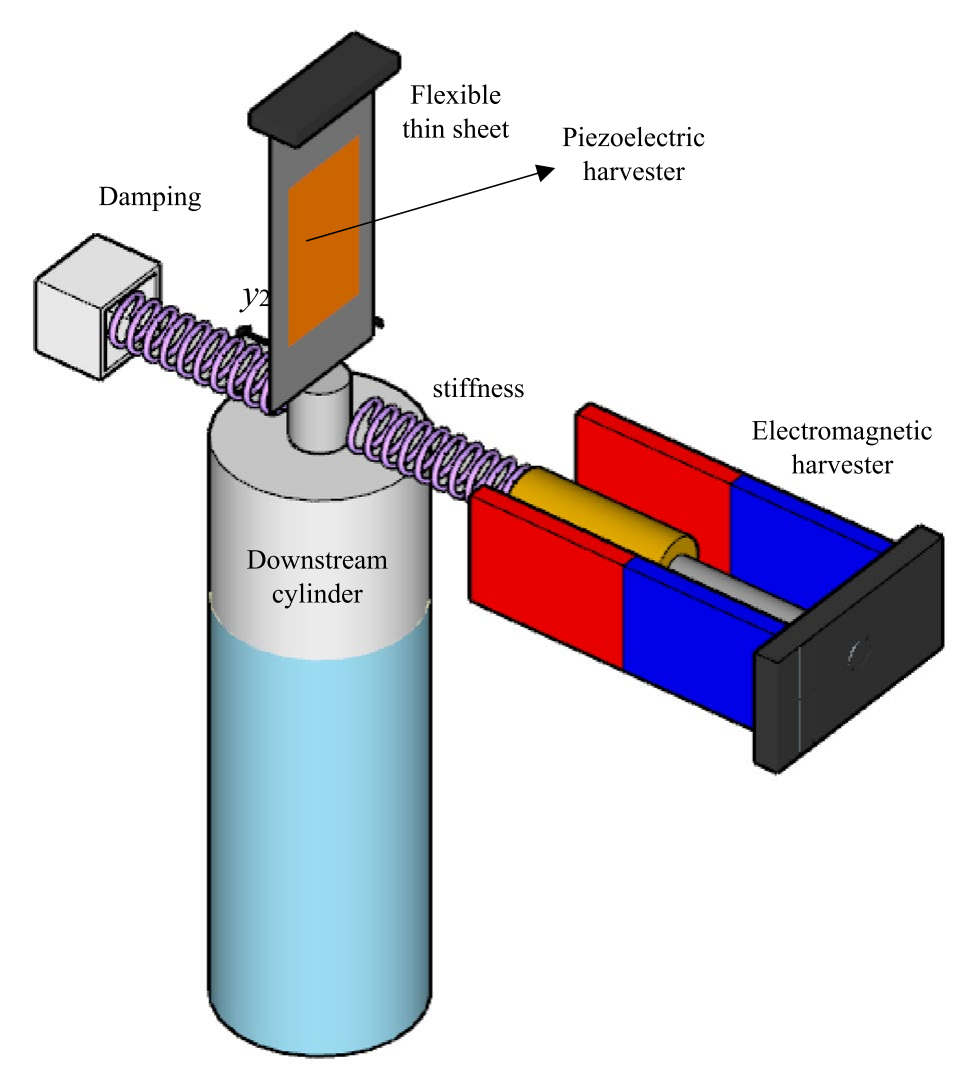
Schematic of the hybrid electromagnetic–piezoelectric tandem harvester (HEPT-H). Both piezoelectric and electromagnetic transducers are coupled to the downstream cylinder, enabling combined conversion of mechanical oscillations into electrical energy through voltage and electrical current outputs.

This section introduces a HEPT-H system that integrates both electromagnetic and piezoelectric harvesters, coupled with the FIO oscillator (Fig. 4). It represents the first investigation of the combined solid–fluid–piezoelectric–electromagnetic coupling within an FIO-based framework. The equations are defined as:

Downstream cylinder (VIV + galloping + piezoelectric + electromagnetic coupling):

27 $$\:{\ddot{y}}_{2}+\left(2\xi\:+\frac{{C}_{D2}}{2\mu\:}\sqrt{\frac{{{{X}^{2}U}^{*}}^{2}}{4{\pi\:}^{2}}+{{\dot{y}}_{2}}^{2}}\right){\dot{y}}_{2}+{y}_{2}-{v}_{p}-{i}_{e}=\frac{{{U}^{*}}^{2}{C}_{L12}}{8{\pi\:}^{2}\mu\:}+\left(\sqrt{\frac{{{{X}^{2}U}^{*}}^{2}}{4{\pi\:}^{2}}+{{\dot{y}}_{2}}^{2}}\right)\frac{{U}^{*}{C}_{L02}}{8\pi\:\mu\:}{q}_{2}$$

28 $$\:{\ddot{q}}_{2}+{\in\:}_{2}{St}_{2}{U}^{*}X\left({{q}_{2}}^{2}-1\right){\dot{q}}_{2}+{\left({St}_{2}{U}^{*}X\right)}^{2}{q}_{2}={A}_{2}{\ddot{y}}_{2}$$

29 $$\:{\dot{v}}_{p}+{\sigma\:}_{2,p}{v}_{p}+{\sigma\:}_{1,p}{\dot{y}}_{2}=0$$

30 $$\:\frac{d{i}_{e}}{dt}+{\sigma\:}_{2,e}{i}_{e}+{\sigma\:}_{1,e}{\dot{y}}_{2}=0$$

The mathematical formulation governing the hybrid piezoelectric-electromagnetic energy harvester coupled with an isolated cylinder (HEPI-H) can be written as follows:

31 $$\:{\ddot{y}}_{1}+\left(2\xi\:+\frac{{C}_{D1}}{2\mu\:}\sqrt{\frac{{{U}^{*}}^{2}}{4{\pi\:}^{2}}+{{\dot{y}}_{1}}^{2}}\right){\dot{y}}_{1}+{y}_{1}-{v}_{p}-{i}_{e}=\left(\sqrt{\frac{{{U}^{*}}^{2}}{4{\pi\:}^{2}}+{{\dot{y}}_{1}}^{2}}\right)\frac{{U}^{*}{C}_{L01}}{8\pi\:\mu\:}{q}_{1}$$

32 $$\:{\ddot{q}}_{1}+{\in\:}_{1}{St}_{1}{U}^{*}\left({{q}_{1}}^{2}-1\right){\dot{q}}_{1}+{\left({St}_{1}{U}^{*}\right)}^{2}{q}_{1}={A}_{1}{\ddot{y}}_{1}$$

33 $$\:{\dot{v}}_{p}+{\sigma\:}_{2,p}{v}_{p}+{\sigma\:}_{1,p}{\dot{y}}_{2}=0$$

34 $$\:\frac{d{i}_{e}}{dt}+{\sigma\:}_{2,e}{i}_{e}+{\sigma\:}_{1,e}{\dot{y}}_{2}=0$$

The damping induced by the hybrid piezoelectric-electromagnetic subsystem comprises three components: mechanical damping from the pulley–belt transmission, piezoelectric damping (C_pz_), and electromagnetic damping (*C*_em_​). The former is embedded in the system identification through the damping ratio ($\:\xi\:$), while the latter (*C*_em_​ and C_pz_) represents a virtual damping effect due to the electromotive force generated during power conversion. These additional terms are inherently incorporated into the governing dynamics via the coupled electromagnetic equations (Eqs. (29) and (33)) and the coupled piezoelectric equations (Eqs. (30) and (34)).

### Numerical procedure

The system of coupled nonlinear differential equations is solved using MATLAB’s ode45 solver, which implements a variable-step 8th to 10th -order Runge–Kutta integration scheme. The simulations are initialized with stationary cylinders and limit-cycle conditions for the wake oscillators. Transient behavior is discarded, and steady-state solutions are extracted for post-processing. Harmonic response amplitudes (120s with a frequency of 0.01) are computed based on root-mean-square values over the steady portion of the time series.

For the isolated cylinder configuration without any energy harvester, Eqs. (5) and (6) were applied. For free–free cylinders without any energy harvester, Eqs. (5)–(8) were employed. For configurations incorporating a piezoelectric harvester (PZT-H), Eqs. (5), (6), and (9)–(11) were used, while for the PZI-H system, Eqs. (15)–(17) were considered. In the case of electromagnetic harvesters (EMT-H), Eqs. (5), (6), and (18)–(20) were utilized, whereas for the EMI-H system, Eqs. (24)–(26) were applied. Finally, for hybrid piezoelectric–electromagnetic harvesters (HEPT-H), Eqs. (5), (6), and (27)–(30) were used, and for the HEPI-H configuration, Eqs. (31)–(34) were adopted. The parameters adopted for the FIO systems are summarized in Table 1. The electrical circuit parameters used in this study were adopted from previously published works that reported experimentally optimized or validated values for similar piezoelectric and electromagnetic systems. These parameters, therefore, represent near-optimal operating conditions commonly observed in FIO-based energy harvesting systems^2,4,39,47,69,70^. Although detailed circuit optimization is beyond the current scope, future studies could optimize these electrical parameters to improve the efficiency of the hybrid configuration further.

The coupled nonlinear differential equations governing the fluid–structure interaction were solved in MATLAB using the adaptive Runge–Kutta ODE45 solver. Time-step values of Δ*t* = 0.01 were used for final output and post-processing. The total simulation time (*t* = 1200) was sufficient to eliminate transient effects and capture stable periodic responses. This procedure follows the numerical stability and convergence approach described in Soares and Srinil^62^.

**Table 1:** The parameters adopted for the FIO systems.

Parameter		Description	Value	
Structural	*m* ^*^	Mass ratio	2.6	
	*M*	Total effective mass (Kg)	5.85	
	Natural frequency	Natural frequency of cylinders in water (Hz)	0.3	
	$\:\xi\:$	Damping ratio	0.007	
	$\:\mu\:$	The reduced effective mass	2.83	
	*D*	Cylinder diameter (m)	0.05 m	
	*L*	Cylinder length (m)	0.65 m	
Tandem wake oscillator	$\:{\in\:}_{1}$	Empirical wake oscillator coefficient of the upstream cylinder	0.011	
	$\:{A}_{1}$	Empirical wake oscillator coefficient of the upstream cylinder	10	
	*St*_1_, *St*_2_	Strouhal numbers	0.2	
	$\:{C}_{D1}$	Mean drag coefficient of the upstream cylinder	2	
	$\:{C}_{D2}$	Mean drag coefficient of the downstream cylinder	1.2	
	$\:{C}_{L01}$, $\:{C}_{L02}$	Fluctuating lift force coefficient	0.3	
	$\:\lambda\:$	Empirical wake profile coefficient	0.74 *d* ^0.61^	
	$\:\alpha\:$	Empirical wake profile coefficients	0.53 *d* ^0.17^	
	$\:\beta\:$	Empirical wake profile coefficients	$\:\gamma\:<0$	0.15
			$\:\gamma\:=0$	0
			$\:\gamma\:>0$	-0.15
	$\:{\in\:}_{2}$	Empirical wake oscillator coefficient of a downstream cylinder	0.1	
	$\:{A}_{2}$	Empirical wake oscillator coefficient of a downstream cylinder	U^*^ ≤5	25
			U^*^ >5	$\:\left(3.206{e}^{-0.45d}+1.163{e}^{-0.45d}\right)\left[\frac{43.59{{U}^{*}}^{2}-626{U}^{*}+2669}{{{U}^{*}}^{2}-5.29{U}^{*}+19.82}\right]$
Isolated cylinder wake oscillator	$\:{C}_{L01}$	Fluctuating lift force coefficient	0.3842	
	$\:{C}_{D1}$	Mean drag coefficient of the isolated cylinder	1.1856	
	*St*_1_	Strouhal numbers	0.1932	
	$\:{\in\:}_{1}$	Empirical wake oscillator coefficient of the isolated cylinder	U^*^ <6.5	0.05
			U^*^ ≥6.5	0.7
	$\:{A}_{1}$	Empirical wake oscillator coefficient of the isolated cylinder	U^*^ <6.5	4
			U^*^ ≥6.5	12
Electromagnetic harvester	$\:{\theta\:}_{e}$	Electromagnetic–mechanical coupling constant (N/V)	17.5	
	$\:{L}_{c}$	Electromagnetic coil inductance (H)	0.0656	
	$\:{R}_{e}$	Load resistance (EMT-H circuit) (Ω)	100	
Piezoelectric harvester	$\:{\theta\:}_{p}$	Piezoelectric–mechanical coupling constant (N/A)	0.004407	
	$\:{C}_{p}$	Piezoelectric capacitance (F)	1.3824 × 10^− 7^	
	$\:{R}_{p}$	Load resistance (PZT-H circuit) (Ω)	130,000	

The piecewise parameter setting adopted in this study ($\:{U}^{*}$≤5 and $\:{U}^{*}$>5) is motivated by the modeling framework introduced by Ogink and Metrikine^71^. This study showed that the conventional van der Pol oscillator could not accurately reproduce the complete FIO response of a cylinder using a single parameter set. By independently validating the lower and upper branches of the initial response curve, they successfully extended the applicability of the wake oscillator model to a broader range of reduced velocities and structural configurations. Following this pioneering concept, several subsequent studies further developed and refined the van der Pol–based formulations^2,3,72^ to improve their ability to predict different dynamic regimes of flow-induced oscillations across a wider range of mass–damping and flow conditions. Later, Soares and Srinil^62^ adopted a similar approach and explicitly separated the galloping and upper branch regimes from the initial (lock-in) branch, thereby improving the model’s capability to represent nonlinear wake–body coupling effects in tandem-cylinder configurations. Accordingly, in this study, the threshold value of $\:{U}^{*}$=5 is adopted to delineate the VIV-dominated regime from the galloping-dominated regime, consistent with previous experimental and theoretical findings. This piecewise formulation is physically motivated by the well-known separation between the VIV-dominated initial and upper branches and the galloping-dominated post–lock-in regime. For U^*^≤ 5, the hydrodynamic forcing is primarily governed by periodic vortex shedding, and the van der Pol oscillator parameters reproduce the lock-in dynamics. For U^*^> 5, wake-induced changes in mean lift and shear-layer asymmetry increase the negative aerodynamic damping, enabling galloping to develop. Using distinct parameter sets, therefore, allows the model to accurately capture the transition from vortex-synchronized oscillations to the large-amplitude, self-excited galloping response observed in experiments.

## Validation

To verify the accuracy and reliability of the proposed HEPT-H wake-deficit oscillator model, simulation results were compared against experimental and numerical findings from prior benchmark studies, including those by Assi^64^, Pereira et al.^73^, Soares and Srinil^62^, and Lin et al.^66^. The validation was conducted for spacing ratios *d* = 4, 8, and 20, covering conditions from strong wake interference to near-isolated flow regimes.

**Fig. 5:**
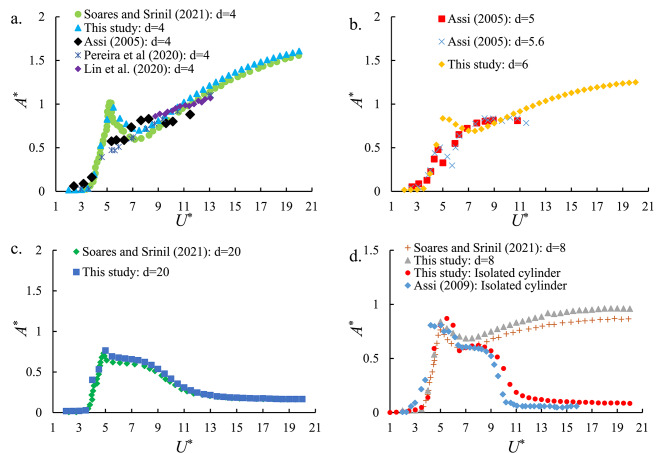
*A* ^*^ versus U^*^ for the downstream cylinder at various spacing ratios and the isolated cylinder. Comparison between the present model and experimental/numerical data. (Assi^64,74^, Pereira et al.^73^, Soares and Srinil^62^, and Lin et al.^66^) validates the ability of the proposed model to reproduce VIV and galloping regimes.

Fig. 5 presents the normalized cross-flow amplitude response (A^*^=displacement/*D*) versus the U^*^ for the downstream cylinder. The comparison shows that the present model reproduces key characteristics observed in previous works, including: Lock-in regions for *d* = 4, *d* = 8, and *d* = 20, where the oscillation frequency synchronizes with the vortex shedding frequency, yielding amplitude peaks consistent with experimental data. Furthermore, vibration onset at lower U^*^ and galloping onset at higher U^*^ for *d* = 4, *d* = 6, *d* = 8, and *d* = 20, in agreement with the trends reported by Soares and Srinil^62^. The isolated cylinder validation also exhibits close agreement with the experimental findings of Assi^74^, as illustrated in Fig. 5d.

Moreover, the calculated RMSE values were 0.079, 0.168, 0.176, and 0.081 when compared with the results of Soares and Srinil^62^, Assi^64^, Pereira et al.^73^, Lin et al.^66^, respectively, at *d* = 4. For *d* = 8, the RMSE was 0.089 in comparison with Soares and Srinil^62^, while for *d* = 20, it further decreased to 0.050, indicating closer agreement with their findings. The isolated cylinder case exhibited an RMSE of 0.133 relative to the experimental data of Assi^74^, demonstrating good model accuracy.

Overall, the close match in both amplitude trends and oscillation characteristics confirms that the proposed coupled oscillator framework accurately captures the dominant fluid–structure interaction mechanisms and can be reliably extended to model hybrid energy harvesting scenarios.

**Fig. 6:**
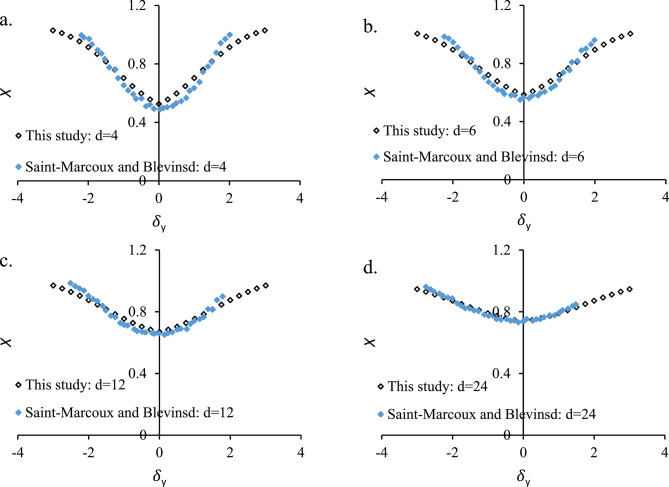
*X* versus $\:{\delta\:}_{y}$ comparison of the wake oscillator model with experimental data of Saint-Marcoux and Blevins^75^ for (**a**) *d* = 4, (**b**) *d* = 6, (**c**) *d* = 12, and (**d**) *d* = 24. The close agreement between the proposed wake-deficit model and experimental data of Saint-Marcoux and Blevins^75^ demonstrates accurate characterization of wake recovery downstream of the upstream cylinder.

As described, Eq. 1 is the mathematical link between the upstream wake flow and the downstream cylinder’s excitation. This equation models how the upstream cylinder’s wake modifies the local flow velocity seen by the downstream cylinder in a tandem configuration. Eq. 1 captures wake deficit (velocity reduction) and wake spreading across the lateral direction, both of which strongly influence the hydrodynamic forces on the downstream cylinder. By providing a quantitative spatial distribution of velocity deficit, it enables to prediction of FIO (both in-line and transverse) on the downstream cylinder. Therefore, by comparing Eq. 1 with experimental data, the model calibrated and validated in offshore, marine, or other engineering contexts where wake interference is important. Therefore without $\:X\left(d,{\delta\:}_{y}\right)$, the FIO component could not be realistically modeled. Fig. 6 indicates *X* versus $\:{\delta\:}_{y}$ comparison of the wake oscillator model with experimental data of Saint-Marcoux and Blevins^75^ for *d* = 4, *d* = 6, *d* = 12, and *d* = 24. The close agreement between the results and experimental data demonstrates strong validation of the proposed model.

## Results and discussion

### FIO results

**Fig. 7:**
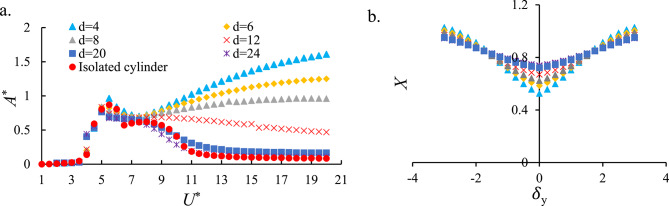
(**a**) A^*^ versus U^*^ for the downstream cylinder and isolated cylinder, and (**b**) normalized wake velocity profile *X* as a function of the *δ*_y_ for spacing ratios d = 4, 6, 8, 12, 20, and 24. Results illustrate the transition from galloping to VIV regimes and the weakening of wake interference with increasing cylinder spacing.

As a first step in this study to characterize the fundamental FIO behavior, the free–free tandem cylinder configuration was initially modeled without incorporating any energy harvester. Fig. 7a presents the variation of A^*^ versus U^*^ for the isolated cylinder and the downstream cylinder at spacing ratios *d* = 4, 6, 8, 12, 20, and 24. The results reveal that increasing the spacing between the two cylinders leads to a reduction in A^*^. This attenuation in amplitude is significant enough to alter the response regime from galloping to VIV. This trend is consistent with previous findings for single cylinders, where the downstream response at *d* = 20 and 24 closely resembles that of an isolated cylinder. The onset of the lock-in range occurs at U^*^=3.5 across all configurations, while galloping initiates at U^*^=8.5 for *d* = 4, 6, 8, and 12. Among these, the configuration with *d* = 4 exhibits the highest amplitude in both the upper branch and galloping regime, with a progressive decrease in amplitude observed as the spacing ratio increases.

The trends in Fig. 7 clearly illustrate the shift from VIV to galloping. For U^*^< 5, all configurations exhibit classic VIV behavior with moderate amplitudes governed by lock-in. As U^*^ exceeds approximately 8–9, the downstream cylinder, particularly for small spacing ratios (*d* = 4–8), enters a galloping regime where the aerodynamic restoring force becomes destabilizing. This produces a rapid growth in amplitude and a sharp increase in the available mechanical energy. For larger spacing ratios (*d* ≥ 20), the wake deficit is weak and the flow approaching the downstream cylinder resembles an isolated cylinder, suppressing galloping and causing VIV to remain the dominant mechanism throughout the entire U^*^ range. These observations confirm the dynamic conditions governing the VIV–galloping transition and identify the dominant instability across spacing ratios.

Fig. 7b presents the normalized wake velocity profile (*X*) as a function of the dimensionless displacement difference between two cylinders ($\:{\delta\:}_{y}$)​ for spacing ratios *d* = 4, 6, 8, 12, 20, and 24. At $\:{\delta\:}_{y}$=0, *X* attains its minimum value, corresponding to the maximum velocity deficit directly behind the upstream cylinder. As ∣$\:{\delta\:}_{y}$∣ increases, *X* approaches unity, indicating that the wake influence diminishes and the flow velocity recovers to the free-stream condition. For *d* = 4, the *X* is relatively narrow, and the centerline velocity deficit is most noticeable (smallest χ_min_ ​). In this case, χ ≈ 1 is reached at a smaller ∣$\:{\delta\:}_{y}$∣, with steeper lateral gradients that signify stronger velocity variation across the wake. With increasing spacing ratios (*d*), the *X* becomes wider and more diffused (associated with a larger *λ*), and *X*_min_​ moves closer to unity, reflecting a weaker deficit. Wider *d* also produces a more gradual variation of *X* with ∣$\:{\delta\:}_{y}$∣, implying that the downstream cylinder is subjected to a milder wake effect.

### Piezoelectric energy harvester (PZT-H)

As mentioned before, for solid-fluid-piezoelectric coupling of the FIO system, Eqs. (5), (6), and (9)–(11) were considered. For investigating HEPT-H output power ($\:{P}_{po}$), after calculating $\:{V}_{p}$ from Eq. 12, $\:{P}_{po}$ calculated based on Ohm’s law as follows^2^:

35 $$\:{P}_{po}=\frac{{{V}_{p}}^{2}}{{R}_{p}}$$

The input mechanical power in this study was derived from the fluid kinetic energy flux acting on the effective cross-sectional area of the downstream cylinder^1^. Input power and energy efficiency could be calculated as follows:

36 $$\:Input\:power={C}_{\mathrm{B}\mathrm{e}\mathrm{t}\mathrm{z}}\times\:\:\rho\:{U}^{3}\left(D+2{y}_{2,\mathrm{m}\mathrm{a}\mathrm{x}}\right)L$$

37 $$\:\eta\:=\frac{{P}_{po}}{Input\:power}$$

where $\:{C}_{\mathrm{B}\mathrm{e}\mathrm{t}\mathrm{z}}$ is the Betz coefficient^3^ ($\:\frac{16}{27}$), $\:\rho\:$ is water density, $\:U$ is flow velocity and $\:{y}_{2,\mathrm{m}\mathrm{a}\mathrm{x}}$ is maximum of $\:{y}_{2}$. The $\:{C}_{\mathrm{B}\mathrm{e}\mathrm{t}\mathrm{z}}$ represents the theoretical maximum fraction of kinetic energy that can be extracted from an open, steady flow by any ideal energy conversion device^3^.

**Fig. 8:**
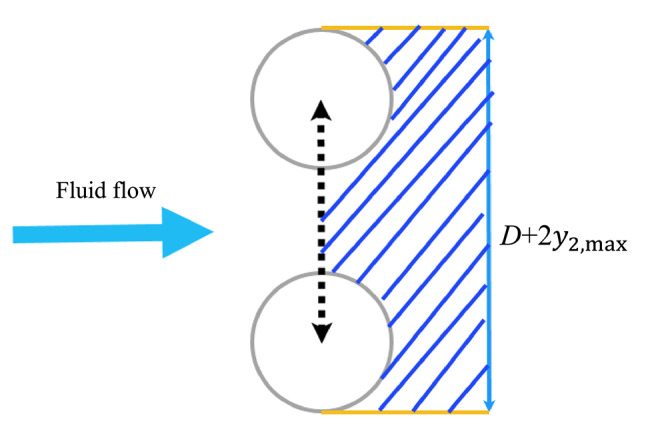
Effective frontal area used in input power calculation.

As shown in Fig. 8, the effective frontal area $\:\left(D+2{y}_{2,\mathrm{m}\mathrm{a}\mathrm{x}}\right)L$ represents the swept area intercepted by the oscillating body in the plane perpendicular to the incoming flow. This is a common approximation in studies of energy harvesting from FIO, where the cross-flow motion expands the effective inflow area beyond the static projected area *DL*.

Physically, during large-amplitude oscillations, the body displaces transversely from −$\:{y}_{2,\mathrm{m}\mathrm{a}\mathrm{x}}$​ to +$\:{y}_{2,\mathrm{m}\mathrm{a}\mathrm{x}}$​, sweeping a total distance of 2$\:{y}_{2,\:\mathrm{m}\mathrm{a}\mathrm{x}}$​ in the cross-flow direction. Adding the body’s inherent diameter *D* yields an effective width of $\:D+2{y}_{2,\mathrm{m}\mathrm{a}\mathrm{x}}$​, which accounts for the broader stream tube of fluid with which the body interacts. This mimics the concept of a turbine’s rotor swept area, capturing the increased volume of fluid momentum and kinetic energy available for extraction due to the dynamic motion, rather than just the stationary geometry.

This approximation directly relates to the actual fluid energy input by providing a basis for estimating the theoretical available power from the flow (Eq. 36). The harvested or input energy during FIO can then be normalized against this value to compute $\:\eta\:$, offering a meaningful metric for large-amplitude regimes where quasi-static assumptions (fixed *DL*) underestimate the $\:\eta\:$. Without this adjustment, the model’s predictions would not align with observed power scaling in high-amplitude FIO, as the oscillation enables energy capture from a wider effective frontal region.

**Fig. 9:**
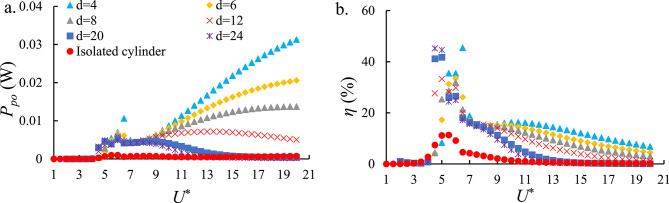
(**a**) The output power of the PZT-H and PZI-H of the FIO-based system and (**b**) the $\:\eta\:$ of the PZT-H and PZI-H of the FIO-based systems versus U^*^. Smaller spacing ratios enhance near-wake coupling, leading to higher power and efficiency in galloping-dominated regimes.

Fig. 9a illustrates the variation of $\:{P}_{po}$​ for downstream cylinders with spacing ratios ranging from *d* = 4 to *d* = 24. The results indicate a general decreasing trend of $\:{P}_{po}$​ as *d* increases. Energy harvesting through galloping is observed at *d* = 4, 6, 8, 12, and 24, suggesting that vortex-induced synchronization persists across multiple spacing conditions. Among these cases, the downstream cylinder at *d* = 4 exhibits the highest $\:{P}_{po}$​ in both the upper and galloping branches, which can be attributed to the strong synchronization between the vortices shed from the upstream and downstream cylinders. This finding highlights the dominant role of near-wake interference at small spacing ratios in enhancing power output. A comparison between the average and maximum values of $\:{P}_{po}$​ for tandem and isolated cylinder configurations indicates that employing a tandem arrangement can enhance both the average and maximum $\:{P}_{po}$​ by approximately 93%.

Fig. 9b presents the efficiency *η* of downstream cylinders for the same range of spacing ratios. The maximum efficiency is observed at *d* = 4 in the upper branch at higher U^*^, which is consistent with the behavior of $\:{P}_{po}$​. However, unlike $\:{P}_{po}$​, the cases of *d* = 20 and *d* = 24 display higher efficiencies in the upper branch compared to intermediate spacing ratios of *d* = 6 to *d* = 12. This trend suggests that at wider spacing ratios, partial recovery of flow coherence may enhance energy conversion efficiency despite the reduced power output. Nevertheless, as U^*^ increases, the efficiency for *d* = 20 and *d* = 24 declines sharply, eventually becoming the lowest among all spacing ratios. This behavior highlights the balance between vortex interactions caused by cylinder spacing and the ability to maintain performance at higher reduced velocities. The comparison of the average and peak *η* ​ values between tandem and isolated cylinders reveals that the tandem configuration yields an approximately 80% and 71% improvement in the average and peak *η*, ​ respectively. A more detailed comparison of the average and maximum values is provided in the Section on performance comparison of PZT-H, EMT-H, HEPT-H, PZI-H, EMI-H, and HEPI-H Systems.

### Electromagnetic energy harvester (EMT-H)

As previously stated, for solid-fluid-electromagnetic coupling of the FIO system, Eqs. (5), (6), and (15)–(17) were considered. For calculating the EMT-H system output power ($\:{P}_{eo}$), after measuring $\:{I}_{e}$ from Eq. 21, $\:{P}_{eo}$ calculated based on Ohm’s law as follows^4^:

38 $$\:{P}_{eo}={{I}_{e}}^{2}{R}_{e}$$

Energy efficiency could be calculated as follows^4^:

39 $$\eta = \frac{{P_{{eo}} }}{{Input~power}}$$

**Fig. 10:**
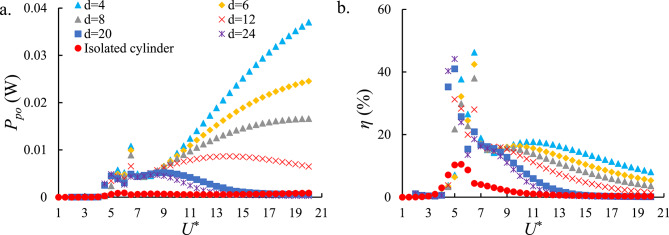
(**a**) The output power of the EMT-H and EMI-H of the FIO-based systems and (**b**) the $\:\eta\:$ of the EMT-H and EMI-H of the FIO-based systems versus U^*^. The trends resemble those of the piezoelectric harvester, with peak power occurring at strong wake-interference conditions (low *d*).

Fig. 10a shows the variation of $\:{P}_{eo}$​ for downstream cylinders with spacing ratios ranging from *d* = 4 to *d* = 24. The results for the EMT-H also indicate a general decreasing trend of $\:{P}_{eo}$​ as *d* increases, these results are similar to the observations reported earlier for the PZT-H. Energy harvesting through galloping is observed for *d* = 4, 6, 8, 12, and 24, suggesting that vortex-induced synchronization persists across multiple spacing conditions. Among these cases, the downstream cylinder at *d* = 4 exhibits the highest $\:{P}_{eo}$​ in both the upper and galloping branches, which can be attributed to strong synchronization between the vortices shed from the upstream and downstream cylinders. Furthermore, the values of $\:{P}_{eo}$​ are comparable to those of $\:{P}_{po}$​, indicating that EMT-H and PZT-H transduction mechanisms possess similar potential for energy harvesting under these flow conditions. Also, the tandem configuration enhanced the average and peak $\:{P}_{eo}$ by about 93% and 94%, respectively, compared to the isolated cylinder.

Fig. 10b demonstrates the *η* of downstream cylinders for the same range of spacing ratios. The maximum efficiency is observed for *d* = 4, 24, 6, and 20, respectively, in the upper branch. Notably, the cases of *d* = 20 and *d* = 24 display higher efficiencies in the upper branch compared to the intermediate spacing ratios of *d* = 8 and *d* = 12. This trend suggests that at wider *d*, partial recovery of flow coherence may enhance energy conversion efficiency despite the overall reduction in power output. However, as U^*^ increases, the efficiencies for *d* = 20 and *d* = 24 decline sharply, ultimately becoming the lowest among all spacing ratios. Moreover, the tandem configuration demonstrated notable performance gains, with the average and peak *η* increasing by approximately 81% and 74%, respectively, relative to the isolated cylinder. A detailed assessment of the average and maximum values is presented in the Section on performance comparison of PZT-H, EMT-H, and HEPT-H Systems.

### Hybrid electromagnet-piezoelectric energy harvester coupled with tandem cylinders (HEPT-H)

For the HEPT-H energy harvesting system, the output powers of the piezoelectric and electromagnetic generators are calculated using Eqs. 35 and 38, respectively. The total output power of the HEPT-H system ($\:{P}_{total}$) is obtained by summing the individual contributions, $\:{P}_{eo}$ and $\:{P}_{po}$. The energy efficiency is then calculated as follows:

40 $$P_{{total}} = P_{{eo}} + P_{{po}}$$

41 $$\eta = \frac{{P_{{total}} }}{{Input~power}}$$

**Fig. 11:**
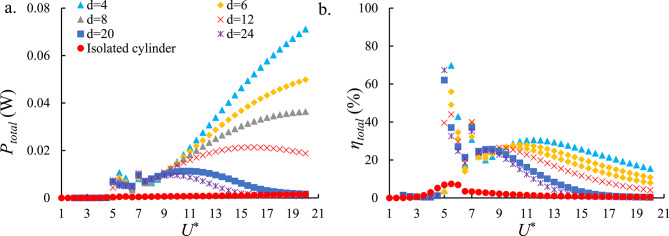
(**a**) The total output power of the HEPT-H and HEPI-H systems and (**b**) the $\:\eta\:$ of the HEPT-H and HEPI-H systems versus U^*^. The hybrid configuration combines piezoelectric and electromagnetic mechanisms, achieving nearly double the power and ~ 20% higher efficiency compared with single harvesters, especially in galloping regimes at *d* = 4.

Fig. 11a indicates the total output power of the HEPT-H system. The results exhibit trends similar to those observed for the individual generators in the VIV-based system, where increasing the spacing between cylinders leads to a reduction in output power. A comparison of Fig. 11a with Figs. 9a and 10a reveal that the output power in the upper branch remains comparable across all cases. This behavior can be attributed to the additional damping introduced by the combined action of PZT-H and EMT-H. In contrast, within the galloping branch, the HEPT-H configuration nearly doubles the harvested power compared to the single-harvester cases. This finding highlights the significant potential of galloping-induced vibrations to support HEPT-H simultaneously. Indeed, the HEPT-H system demonstrates strong capability for enhancing energy extraction in galloping-dominated regimes. When compared to the isolated cylinder, the tandem arrangement exhibited significant improvements in both the average and peak $\:{P}_{total}$, achieving approximately 95% increases, respectively.

Fig. 11b illustrates the $\:\eta\:$ of the HEPT-H system. The downstream cylinder at *d* = 4 continues to achieve the highest efficiency, consistent with previous observations. Moreover, the efficiency of the HEPT-H system is approximately 20% higher than that of either individual harvester. The observed improvement indicates that although the HEPT-H system implies additional damping that reduces the available input power, the FIO-based HEPT-H system still can capture a larger fraction of this power, thereby enhancing the overall energy conversion efficiency. Comparative analysis of the tandem and isolated cylinder configurations reveals that the tandem arrangement produces approximately 89% and 87% improvements in the average and peak $\:\eta\:$, respectively, highlighting the efficiency advantages of hydrodynamic interaction between the cylinders.

Galloping and VIV are fundamentally different mechanisms. VIV is driven by periodic vortex shedding (Kármán street) that produces a narrow-band, frequency-locked forcing; it typically yields moderate amplitudes when the structure is locked to the shedding frequency (lock-in). By contrast, galloping is a self-excited instability that arises when the instantaneous aerodynamic transverse force has a component whose sign produces negative aerodynamic damping, that is, aerodynamic forces feed energy into the oscillator as displacement grows. Physically, galloping occurs when flow separation and asymmetric shear layers around the displaced body produce a displacement-dependent lift that reinforces motion; because this is not frequency-locked to a single vortex shedding frequency, galloping can produce much larger amplitudes and therefore much larger extractable mechanical power^9^.

In tandem arrangements, the upstream wake modifies local velocity, separation points, and unsteady pressure on the downstream cylinder. For small spacing ratios (near-wake interference: d = 4), the wake deficit is strong and lateral gradients are steep (see *X* and discussion in Fig. 7), which enhances forcing asymmetries and can move the system into galloping at lower reduced velocities. The wake also modifies the effective Strouhal/shedding patterns (2Single, 2Pairs, 2Pairs + 2Single modes have been observed in similar tandem studies^15,29,41,43,44,76^, so the combined effect of wake-induced mean force changes and altered vortex interactions favors the large amplitude, broadband response characteristic of galloping in our tandem cases and consequently, the harvested power is substantially increased. (Figs. 7, 8, 9, 10 and 11).

**Fig. 12:**
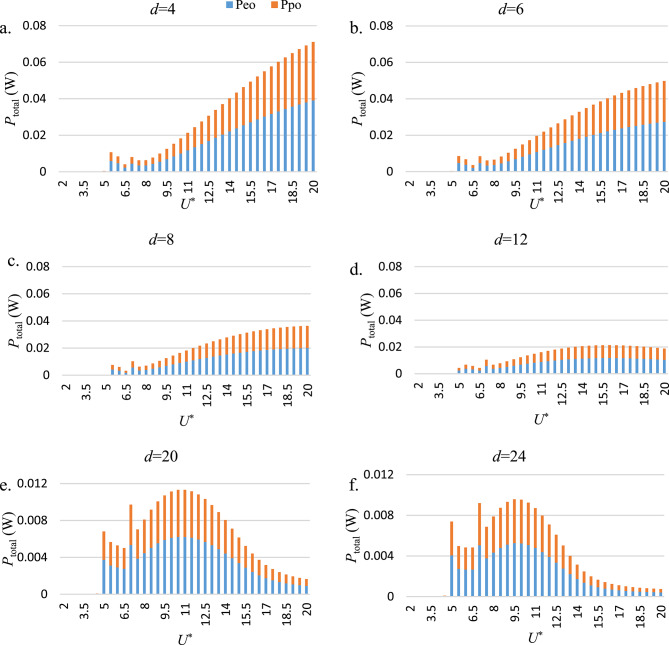
The $\:{P}_{total}$ versus U^*^, separated into the contributions from the electromagnetic ($\:{P}_{eo}$​) and piezoelectric ($\:{P}_{po}$​) harvesters.

Fig. 12 illustrates the portion of generated power each harvester ($\:{P}_{eo}$ and $\:{P}_{po}$), separately. This figure is used to evaluate the relative performance of each generator within the HEPT-H system across different *d*. The results indicate that, except for the galloping branch, both harvesters contribute nearly equal amounts of power. However, in the galloping branch, the electromagnetic harvester exhibits slightly superior performance compared to the piezoelectric harvester. It is noteworthy that the relative contribution of each harvester in the HEPT-H configuration may vary depending on the electrical parameters of their respective circuits. A systematic investigation into circuit optimization could therefore serve as an important direction for future work to enhance HEPT-H system performance. These findings emphasize the critical role of galloping regimes in maximizing the potential of HEPT-H energy harvesting systems.

Beyond numerical validation, the scalability of the HEPT-H system for deployment in marine and riverine environments necessitates careful consideration of several practical factors. Rashki et al.^16^, based on 35 years of real-environment deployments, reported that biofouling can increase surface roughness and subsequently alter hydrodynamic forces over time. In addition, sediment accumulation and corrosion may compromise the long-term durability of both structural and electrical components^2,27,77^. Although detailed field-scale validation lies beyond the scope of the present study, addressing these challenges constitutes a critical direction for future research toward ensuring the reliability and large-scale applicability of the HEPT-H system in natural water flows.

From a synchronization/phase viewpoint, the two electrical circuits have their own electrical time-scales and therefore impose different phase lags between displacement and the electrical response. These phase lags alter the relative phase between the hydrodynamic forcing (lift/flow excitation) and the structural motion; because power extraction is proportional to the in-phase component of force×velocity, circuit time constants and load tuning directly influence how much of the mechanical energy is converted in each channel. In practice (This study), both harvesters contribute roughly equally outside galloping, but in the galloping branch, the electromagnetic subsystem provides a slightly larger share consistent with the electromagnetic circuit time constants and coupling coefficients used here (Fig. 12). This demonstrates that circuit tuning (load/resonance) is a practical lever for shifting power partitioning in hybrid systems.

Finally, energy transfer in the hybrid configuration is the algebraic sum of two conversion paths (piezoelectric voltage generation and electromagnetic current generation). Hybridization typically increases the total extracted power because the two transducers tap the same structural motion with different couplings and electrical boundary conditions; however, the extra extraction also increases net effective damping, which can reduce available mechanical input power if over-damped. In our tandem cases, the hybrid system nevertheless increases total captured power (nearly doubles in galloping) and improves efficiency by ≈ 20% (HEPT-H vs. single harvesters) because galloping provides sufficiently large mechanical input to overcome the added damping (Fig. 11).

### Performance comparison of PZT-H, EMT-H, and HEPT-H systems

To compare the performance of the studied FIO-based energy harvesting systems, four key parameters are considered: the maximum of $\:\eta\:$ ($\:{\eta\:}_{max}$), the maximum output power of each system ($\:{P}_{Max}$), the average of $\:\eta\:$ ($\:{\eta\:}_{avg}$), the average of the output power of each system ($\:{P}_{avg}$). Among these, $\:{\eta\:}_{avg}$ and $\:{P}_{avg}$ are critical indicators of the energy harvesting capability of the FIO-based energy harvesting system. The values of these parameters for each system are summarized in Table 2.

For this purpose, considering three harvester systems (PZT-H, EMT-H, and HEPT-H) and six different cylinder spacing ratios (*d* = 4, 6, 8, 12, 20, and 24), a total of 18 configurations were compared using the TOPSIS method.

To rank the 18 systems’ performance based on these four performance criteria, the Technique for Order of Preference by Similarity to Ideal Solution (TOPSIS) is employed^78,79^. The decision matrix $\:S=\:\left\{{s}_{\mathrm{i}\mathrm{j}}|i=1.2.\dots\:.m;j=1.2.\dots\:.n\right\}$ where *n*=4 is the number of criteria including $\:{\eta\:}_{max}$, $\:{P}_{Max}$, $\:{\eta\:}_{avg}$, and $\:{P}_{avg}$and *m*=18 is the number of alternatives, including 18 mentioned systems, which is constructed from the data in Table 2. The matrix $\:S$ is then normalized using Eq. 42:

42 $$\:NS={\left({ns}_{ij}\right)}_{m\times\:n}=\frac{{s}_{ij}*{w}_{j}}{\sqrt{\sum\:_{i=1}^{m}{s}_{ij}^{2}}}$$

where $\:NS$ is the normalized matrix derived from $\:S$, and $\:{w}_{j}$ represents the weight assigned to each criterion. In this study, the weights are assigned as follows: $\:w=0.15$ for both $\:{\eta\:}_{avg}$ and $\:{P}_{avg}$, $\:w=0.35$^40^ for both $\:{\eta\:}_{max}$ and $\:{P}_{max}$. A higher weight was assigned to the maximum values ($\:{\eta\:}_{max}$ and $\:{P}_{Max}$) compared to the average values ($\:{\eta\:}_{avg}$and $\:{P}_{avg}$), as peak performance is considered a critical indicator of the system’s capability to exploit extreme flow conditions and achieve maximum energy harvesting efficiency. The weighting factors in the TOPSIS analysis were selected considering the practical relevance of each performance indicator. In flow-induced oscillation energy-harvesting systems, the maximum output power has greater importance for long-term operational efficiency, as the system can be tuned to operate near a nearly constant flow velocity corresponding to its maximum response. Accordingly, a higher weight was assigned to the maximum performance criteria. Additional tests with equal and alternative weighting distributions showed that the top three rankings remained unchanged, and the remaining alternatives varied by at most one rank, confirming the robustness of the adopted weighting scheme.

Using these weights, the weighted normalized decision matrix is constructed according to Eq. 42. Subsequently, the positive ideal solution ($\:ns$^+^) and the negative ideal solution ($\:ns$^−^) for each criterion are identified. For the FIO-based systems listed in Table 2, the Euclidean distances of each alternative from the ideal and non-ideal solutions are then calculated using Eqs. 43 and 44, respectively.

43 $$\:{v}_{i}^{+}={\left[\sum\:_{j=1}^{m}{({ns}_{ij}-{ns}_{j}^{+})}^{2}\right]}^{0.5}$$

44 $$\:{v}_{i}^{-}={\left[\sum\:_{j=1}^{m}{({ns}_{ij}-{ns}_{j}^{-})}^{2}\right]}^{0.5}$$

Finally, the FIO-based systems are ranked based on their relative closeness to the ideal solution, calculated using Eq. 45. In Table 2, a higher *J*_i_ value indicates greater proximity to the optimal condition, reflecting superior overall performance of the FIO-based systems.

45 $$\:{J}_{i}=\frac{{v}_{i}^{-}}{{v}_{i}^{+}+\:{v}_{i}^{-}}$$

In Table 2, the performance of piezoelectric (PZT-H), electromagnetic (EMT-H), and hybrid harvesters in tandem cylinder configurations (HEPT-H) is evaluated using the TOPSIS method. The criteria considered include $\:{\eta\:}_{max}$, $\:{P}_{Max}$, $\:{\eta\:}_{avg}$, and $\:{P}_{avg}$. The parameter *d* represents the spacing between tandem cylinders. The normalized TOPSIS index ($\:{J}_{i}$​) and the corresponding rank are also reported, providing a comprehensive comparison of system performance across different harvester types and spacing ratios.

The TOPSIS evaluation reveals that the HEPT-H at *d* = 4 achieves the best overall performance, with the highest score ($\:{J}_{i}$​=1.0) and rank 1. This configuration attains a maximum power output of 0.0711 W, maximum efficiency of 69.78%, and the highest average efficiency ($\:{\eta\:}_{avg}$​=21.67%), clearly outperforming all other cases. HEPT-H systems at *d* = 6 and *d* = 8 follow closely, occupying ranks 2 and 3, respectively. This highlights the significant advantage of hybrid configurations, particularly at closer spacing ratios where strong vortex-induced interactions enhance energy harvesting.

Among the EMT-H and PZT-H systems, the EMT-H at *d* = 4 performs best ($\:{J}_{i}$​​=0.541, rank 4), slightly outperforming its PZT-H counterpart at the same spacing ($\:{J}_{i}$=0.477, rank 5). Both cases demonstrate the importance of near-wake interference at small spacing ratios in maximizing performance. In contrast, for wider spacing ratios (*d* = 20 and *d* = 24), both PZT-H and EMT-H show weak performance with $\:{J}_{i}$​<0.25, underscoring the decline in energy harvesting capability as vortex synchronization diminishes.

Interestingly, the HEPT-H at wider spacing ratios (*d* = 20 and *d* = 24) still exhibits relatively higher efficiencies ($\:{\eta\:}_{max}$=62.1% and 67.32%, respectively), despite their lower absolute power output. This suggests that HEPT-H systems retain the capacity to convert a larger fraction of the available flow energy into usable power, even under conditions where vortex-induced oscillations are weaker.

The inclusion of isolated harvesters (PZI-H, EMI-H, and HEPI-H) provides a useful baseline for comparison. These single-cylinder systems yield notably lower $\:{P}_{max}$, and *J*_i_ ​indices below 0.03. Their efficiencies remain under 12%, confirming the limited capability of standalone harvesters in exploiting flow-induced oscillations effectively. Among them, the piezoelectric harvester (PZI-H) achieves the $\:{\eta\:}_{max}$, followed by the electromagnetic (EMI-H) and hybrid electromagnetic–piezoelectric (HEPI-H) cases. The comparatively poor performance of HEPI-H suggests that hybridization without hydrodynamic coupling (as in the tandem configuration) does not inherently improve performance; instead, it adds extra damping, which suppresses oscillations and limits energy extraction.

Overall, the TOPSIS evaluation demonstrates that the considered HEPT-H system significantly outperforms single harvester mechanisms across nearly all spacing ratios, particularly in the galloping regime at closer spacing ratios. These findings reinforce the potential of hybrid energy harvesting strategies for maximizing output in FIO-based systems.

**Table 2:** TOPSIS decision matrix for performance ranking of different FIO-based systems and value and weight of parameters for each system.

Criteria	$\:{P}_{max}$ (W)	$\:{\eta\:}_{max}$ (%)	$\:{P}_{avg}$ (W)	$\:{\eta\:}_{avg}$ (%)	J_i_	Rank
Weight System	0.35	0.35	0.15	0.15		
d = 4; PZT-H	0.0313	45.48	0.0135	12.96	0.477	5
d = 6; PZT-H	0.0206	33.47	0.0102	10.9	0.324	10
d = 8; PZT-H	0.0137	31.87	0.008	9.782	0.251	12
d = 12; PZT-H	0.0072	33.25	0.0051	8.981	0.202	18
d = 20; PZT-H	0.0052	41.76	0.0021	7.272	0.224	15
d = 24; PZT-H	0.0049	45.2	0.0016	6.742	0.237	13
d = 4; EMT-H	0.037	46.26	0.0154	13.69	0.541	4
d = 6; EMT-H	0.0245	42.45	0.0117	11.66	0.396	6
d = 8; EMT-H	0.0166	38.02	0.0093	10.67	0.303	11
d = 12; EMT-H	0.0086	31.26	0.0059	8.894	0.204	17
d = 20; EMT-H	0.0052	41.02	0.0023	7.287	0.22	16
d = 24; EMT-H	0.0048	44.17	0.0018	6.67	0.232	14
d = 4; HEPT-H	0.0711	69.78	0.0276	21.67	1	1
d = 6; HEPT-H	0.0498	55.97	0.0218	18.63	0.725	2
d = 8; HEPT-H	0.0364	49.13	0.0181	16.87	0.559	3
d = 12; HEPT-H	0.0214	44.02	0.0131	15.57	0.389	7
d = 20; HEPT-H	0.0113	62.1	0.0058	11.99	0.354	9
d = 24; HEPT-H	0.0096	67.32	0.004	10.46	0.358	8
PZI-H	0.001	11.31	0.0005	1.902	0.026	19
EMI-H	0.0009	10.52	0.0005	1.877	0.021	20
HEPI-H	0.0016	7.399	0.0007	1.754	0.008	21

When interpreting the results of VIV experiments and analyzing the associated parameters, it is essential to account for the influence of uncertainties. One primary source of uncertainty in VIV testing arises from the accuracy of free-stream velocity measurements. The velocity of the carriage in each test group is typically estimated by dividing a fixed travel distance of 15 m (approximately half the length of the towing tank) by the corresponding travel time. As indicated by Zeinoddini et al.^80^, this estimation approach introduces minor uncertainty due to potential timing inaccuracies and transient flow effects within the tank. Reported measurements show that the average carriage speed generally deviates within ± 1.5% of the rated speed of the device^80^. Additionally, experimental investigations have shown that the damping ratio may exhibit an approximate deviation of ± 21% from the nominal value^81^.

Further sources of uncertainty are attributed to the electromechanical components of the hybrid energy harvesting system. Previous studies have reported that the piezoelectric^82^ and electromagnetic^83^ couplings contribute uncertainties of approximately ± 1.5% and ± 3%, respectively. According to Hughes and Hase^84^, the propagated uncertainties in displacement amplitude, harvested power, and conversion efficiency are estimated to be around ± 1.76%, ± 6.4%, and ± 6.1% for the EMT-H; ±0.7%, ± 3.6%, and ± 3.7% for the PZT-H; and ± 1.3%, ± 4.4%, and ± 4.0% for the HEPT-H; ±0.6%, ± 5.9%, and ± 6% for the EMI-H; ±1%, ± 3.8%, and ± 3.7% for the PZI-H; and ± 1%, ± 3.7%, and ± 4.0% for the HEPI-H respectively.

## Conclusion

This research investigates the innovative hybrid piezoelectric-electromagnetic harvester coupled with free–free tandem cylinders (HEPT-H) and compares 2 single harvesters with HEPT-H in various spacing ratios. For this purpose, a nonlinear wake-deficit oscillator framework that was successfully developed to model FIO-based free–free tandem cylinders with piezoelectric (PZT-H), electromagnetic (EMT-H), and a new proposed HEPT-H. Validation against experimental and numerical benchmarks confirmed that the proposed model accurately reproduces VIV and galloping characteristics under wake interference regimes. The following conclusions can be drawn:

- Results show that closer spacing ratios (*d* = 4–6) generate stronger wake interactions, leading to higher vibration amplitudes and enhanced power output compared to wider spaces.
- Galloping regimes contribute significantly more to harvested energy than VIV, making them critical for maximizing system performance.
- Both PZT-H and EMT-H achieved comparable performance individually; however, their efficiencies declined at wider spacing ratios (*d* ≥ 20).
- The proposed hybrid harvester demonstrated superior performance, nearly doubling power output in galloping conditions and achieving ~ 20% higher efficiency than the 2 single-harvester systems.
- A multi-criteria decision-making method (TOPSIS), which was employed to rank 18 systems, revealed the hybrid harvester at *d* = 4 as the optimal configuration, with maximum output power of 0.0711 W and energy efficiency of 69.78%.
- HEPT-H systems with different *d* values maintained relatively high efficiencies even at wider spacing ratios, despite reduced absolute power, highlighting their robustness across flow conditions.
- Isolated harvesters (PZI-H, EMI-H, and HEPI-H) exhibit low performance with *J*_i_ ​<0.03 and efficiencies below 12%, confirming the limited capability of standalone systems; among them, PZI-H shows the highest efficiency, while the reduced performance of HEPI-H indicates that hybridization without hydrodynamic coupling introduces additional damping that suppresses oscillations and limits energy extraction.

Overall, the study demonstrates the strong potential of FIO-based hybrid piezoelectric–electromagnetic harvesters for scalable and sustainable hydrokinetic energy conversion. Although the proposed hybrid configuration demonstrates strong potential for efficient energy harvesting through flow-induced oscillations, the findings are based on idealized modeling conditions. The present analysis assumes a uniform steady inflow, rigid cylinders, and two-dimensional hydrodynamic interactions without accounting for turbulence, variable flow direction, or structural flexibility. These simplifications were intentionally made to isolate the fundamental fluid–structure–energy coupling mechanisms. Future work will extend the model to incorporate three-dimensional effects, variable flow conditions, and flexible structural behavior, thereby enhancing its applicability to real hydrokinetic environments.

## Data Availability

All data generated or analyzed during this study are included in this published article.

## List of symbols

FIO: Flow-induced oscillations
VIV: Vortex-induced vibration
PZT-H: Piezoelectric harvester
EMT-H: Electromagnetic harvester
HEPT-H: Hybrid electromagnetic–piezoelectric harvester coupled with tandem cylinders
TOPSIS: Technique for order preference by similarity to ideal solution
*A*^*^: Dimensionless amplitude (displacement/D)
*C*_D_: Drag coefficient
*C*_L_: Lift coefficient
*C*_p_: Piezoelectric capacitance (F)
$$\:{C}_{\mathrm{B}\mathrm{e}\mathrm{t}\mathrm{z}}$$: Betz coefficient
*C*_em_​: Electromagnetic damping coefficient
*C*_pz_: Piezoelectric damping coefficient
*d*: Spacing ratio (d = S/D)
*D*: Cylinder diameter (m)
*i*: Dimensionless electric current (EMT-H)
*I*: Electric current (electromagnetic circuit) (A)
*J*_i_: Normalized TOPSIS performance index
*L*_*c*_: Electromagnetic coil inductance (H)
*L*: Cylinder length (m)
$$\:{m}^{*}$$: Mass ratio
*M*: Total effective mass (kg)
$\:{P}_{po}$: Piezoelectric output harvested power (W)
$\:{P}_{eo}$: Electromagnetic output harvested power (W)
$\:{P}_{total}$: Total output harvested power (W)
*q*_1_, *q*_2_: Wake oscillator variables
*R*_p_: Load resistance (PZT-H circuit) (Ω)
*R*_e_: Load resistance (EMT-H circuit) (Ω)
Re: Reynolds number
St_1_, St_2_: Strouhal numbers (upstream, downstream)
*S*: Center-to-center distance between the cylinders (m)
$$\:{U}^{*}$$: Reduced velocity [U/(f_n_·D)
*U*: Flow velocity (m/s)
*V*: Electric voltage (piezoelectric circuit) (V)
*v*: Dimensionless electric voltage (PZT-H)
*X*: Normalized wake velocity
*y*_1_, *y*_2_: Dimensionless cross-flow displacements (normalized by D)
*λ*: Empirical wake profile coefficient
$\:{\delta\:}_{y}$: Dimensionless staggered position between cylinders
$\:\alpha\:$, $\:\beta\:$: Empirical wake profile coefficients
$\:{\theta\:}_{e}$: Electromagnetic–mechanical coupling constant (N/V)
$\:{\theta\:}_{p}$: Piezoelectric–mechanical coupling constant (N/A)
$\:\omega\:$: Natural angular frequency (rad/s)
*ζ*: Damping ratio
*η*: Energy efficiency (%)
$\:\rho\:$: Fluid density (water) (kg/m^3^)
